# Can a sovereign health research culture bolster Africa‘s involvement in global health?

**DOI:** 10.1080/16549716.2026.2700792

**Published:** 2026-07-13

**Authors:** Cornelius Ewuoso

**Affiliations:** Steve Biko Centre for Bioethics, University of Witwatersrand, Johannesburg, South Africa

**Keywords:** Africa, Sovereignty, Knowledge Production, Participation

## Abstract

Africa stands at a pivotal moment in its history, uniquely positioned to leverage its global medical evidence base to drive health innovation. Yet this potential is stifled by the continent’s limited participation in generating health knowledge. To shape Africa‘s health destiny and address this challenge, the commentary proposes a sovereign health research culture that prioritizes ethics, domestication of research funding, sustainability, and harmonization.

## Paper Context

- **Main findings:** Funding and resource access crises undermine Africa‘s involvement in knowledge generation.
- **Added knowledge:** A bold sovereign health research culture can usefully help in addressing these issues.
- **Global health impact for policy and action:** The contribution prioritizes a sovereign health research culture, and recommends a mechanism for translating it into a practical governance that asserts Africa‘s authority in health research.

## Background

Africa is at a crossroads, uniquely positioned to harness the global medical evidence base (GMEB) to innovate its health-care systems, yet hindered by its marginal contribution to it. This demands a sovereign health research culture (SHRC) that amplifies Africa‘s voice in global health (GH) matters. Health research sovereignty describes Africa‘s authority to set its own health priorities informed by its health challenges, control its health data, oversee collaborations, and transform research insights into tangible benefits for local communities. The culture encompasses the daily practices (e.g. equitably generating/contributing health knowledge) that make this sovereignty real.

Despite being home to 18% of the world‘s population and shouldering 25% of the global disease burden, Africa contributes only 2% to health knowledge generation [1]. The disparity is starkly evident in HIV/AIDS research, where African researchers authored just 21.61% of the global publications, often serving as secondary authors and undermining their intellectual leadership on these health issues in Africa [2,3]. Such inequity breeds epistemic injustice, sidelining African health concerns and resulting in a distorted global medical narrative and ineffective health strategies. Epistemic injustice also manifests in the GH through the underrepresentation of disease burdens and unequal partnerships [4]. For instance, only 3.9% of cardiovascular care trials published in influential medical journals were conducted exclusively in Africa [5], deepening the inadequate representation of African populations in global medical evidence on cardiovascular care.

During a webinar on 28 January 2026, drawing over 180 attendees from diverse backgrounds and featuring two health diplomats from the International Vaccine Institute and Africa CDC, we discussed potential solutions to this pressing issue. While this article is not a consensus statement from the webinar, it draws on important insights that emerged during this engagement to outline a blueprint for cultivating the SHRC that meaningfully addresses Africa‘s limited participation in the GH knowledge-generation landscape and urges diverse stakeholders to take responsibility for advancing health research across the continent.

## Africa’s low involvement in GH

Two prominent intersecting factors – funding and access – that limit Africa‘s involvement in the GMEB emerged during this engagement. While Africa has the personnel to generate valuable health knowledge, it is inhibited by substantial domestic funding shortfalls for health research. The near absence of robust health research funding from local sources compels many researchers to rely heavily on international funding agencies. A 2016/17 study revealed that approximately one-third of health research funding in South Africa emanated from local sources [6]. A staggering 90% of health research funding across the continent relies on foreign donors, with African governments investing less than 1% of their gross domestic product in research and development (R&D) [7]. The situation is further exacerbated by the disproportionate distribution of foreign funding, which largely favours Eastern and Southern African regions with more established research capacities [8]. For instance, HIV/AIDS research is predominantly led by research teams in Kenya, South Africa, and Uganda, showing the focus of resources on the disease burden.

Heavy reliance on foreign donors not only jeopardizes Africa‘s control over health research but also perpetuates an external view of health research in Africa. This sometimes leads to research agendas in Africa that align more with external interests than Africa‘s priorities. For instance, a study in Northern Africa reveals a disconnect between the region‘s disease burden and the focus of health research efforts [9]. The COVID-19 pandemic also highlights the vulnerabilities of a heavy dependence on foreign assistance. The scarcity of vaccines for African populations sparked urgent calls for pharmaceutical sovereignty, advocating for the localization of vaccine research and production to improve availability and pandemic response. This challenge aligns well with the growing concern that health research in GH far too often involves actors, including researchers undertaking studies in locations other than their own and from more powerful backgrounds than those they study [10].

Access also impacts Africa’s contribution to the GMEB. For example, the existing digital divide amplified by the exorbitant cost of internet connectivity, severely affects many peri-urban and rural African communities. These issues restrict access to health research databases and platforms. Furthermore, many health research scientists in Africa encounter substantial obstacles in disseminating their findings in open-access journals due to financial constraints. A study involving researchers from Nigeria, Kenya, and South Africa found that approximately 58.51% of the surveyed researchers reported that the steep article processing fees inhibit their ability to publish their work openly [11]. Infrastructural challenges, including persistent power outages prevalent in countries like Nigeria and South Africa, can exacerbate these issues. For instance, an unstable power supply undermines efforts to reliably and accurately capture, process, and disseminate health information, creating critical gaps in knowledge generation and data.

## Sovereign health research culture (SHRC)

There is an increasing recognition of the need for Africa to reclaim the reins of its health research initiatives. The statistics are telling: health research publications in the WHO African regions surged from 3,623 in 2000 to an impressive 12,709 in 2014 [12]. Health research hubs like the Science for Africa Foundation (SFA) and the African Academy of Sciences (AAS) have emerged as vital forces in this movement, driving funding initiatives that were once the domain of external organizations. These hubs have become pivotal entities in building research and ethics capacities while championing African-led health research initiatives. Additionally, a study predicts that every $1 spent on R&D in Africa could yield a high return of $405 in societal benefits by 2040 [13].

To increase the continent‘s involvement in the GH, what is needed now is to reimagine a health research governance framework that promotes domestic health research funding and equitably facilitates access by health researchers in Africa to the resources necessary to generate and share information. Hence, the SHRC. Instead of externally informed funding agendas, this culture starts with Africa identifying its priorities through – for example – defining its disease burden, research questions, and values [14]. Rather than an absence of external aid or partnerships with foreign agencies, sovereignty is about the terms of these engagements. While external assistance is welcome, Africa‘s priorities/diversities/good/needs should be the foundation for such collaborations. For instance, Africa‘s diverse gene pool offers significant opportunities for global collaborations in pharmacogenomics and precision medicine. However, such collaboration must begin with Africa‘s interest and be safeguarded by ethical guardrails consistent with sovereignty. The proposed SHRC comprises three crucial components that prioritize ethics, domestication of research funding, sustainability, and harmonization.

### Develop clear funding commitments

African governments must meet the African Union‘s (AU) 1% of GDP investment in R&D target to reduce their dependence on external donors. Unfortunately, many African countries, with notable exceptions to Kenya, South Africa, and Egypt, lag significantly behind this goal [15]. To reinforce this commitment, the AU should require member States to present concrete plans and/or tangible evidence of progress toward meeting this target as a prerequisite for leadership roles or membership in key AU councils, like the Peace and Security Council. Furthermore, the AU should establish sovereign (dedicated) health research funds tailored to continental and regional health needs, thereby supporting those member States that struggle to achieve this investment goal. Very essential, accountability measures should accompany these funds, including potential ineligibility for future support for member States that consistently fail to prioritize R&D investment.

Advocacy efforts to prioritize health in national budgets should be recognized and celebrated within the African health community. Health researchers and organizations should be comfortable with leading such advocacy initiatives, including engaging in policy dialogues. Unfortunately, health researchers often shy away from these discussions, leading to a poor translation of valuable research findings into tangible action and policy.

Philanthropic organizations, including affluent Africans, the private sector, and profitable entities within Africa, should also commit to contributing a fraction of their wealth to R&D initiatives. National governments can play a role in this effort by offering tax and debt relief to private donors and entities that support health research, thereby encouraging increased investment in this critical area. They could also explore crowdfunding systems to grow local support for health research.

### Build sustainable health systems

African health departments should transition toward sustainable health systems that – for example – localize pharmaceutical production, including vaccines and essential medicines. As a central concept in SHRC, building sustainable systems can help reduce dependency while creating the space – both technical and fiscal space – to set and lead research agendas locally in the long run. Although mechanisms for realizing this ambitious goal are many, one is a Global South–South partnership designed for capacity building, technology transfer, and sustainable development. Concretely, this may mean engaging in joint ventures, such as creating a shared infrastructure. For instance, China‘s growing interest in investing in health infrastructures or medical technologies in Africa can be a strategic asset for promoting Africa‘s sovereignty if organized around shared medical manufacturing, joint training to retain capacity/workforce in Africa, and transparent governance. Similarly, partnering with India, known for its capacity to produce affordable and high-quality medicines, can significantly improve vaccine access for African nations. However, it is crucial that these collaborations are designed to empower Africa‘s sovereignty, ensuring the region moves toward self-reliance rather than perpetuating dependency.

Another mechanism could be strategic public–private partnerships that engage States, institutions, and private companies to map, train, and retain talent in Africa, improve timely access to vital resources, address critical infrastructure deficits, and maintain cutting-edge facilities, including open data repositories and journals, advanced laboratories, high-speed internet connections, and power supply. This will empower African health researchers to compete globally. The Africa CDC is already making important strides in this direction through its Partnerships for African Vaccine Manufacturing (PAVM), uniting African member States, international organizations, and the private sector to ensure that 60% of the vaccines are locally produced [16]. This model should be replicated across other areas of health research to elevate Africa‘s status as an important global producer of health products and knowledge.

To promote these strategic partnerships, African governments should prioritize local procurement. Imposing solidarity levies on imported health technologies and innovations can further reorient the GH community’s gaze to African initiatives.

### Harmonize health research frameworks

The Africa CDC and AU are uniquely equipped to lead coordinated health research responses during epidemic and non-epidemic emergencies. This role involves working closely with national research ethics committees, regulatory bodies, and health departments to establish a more integrated approach to health research. They can achieve this goal by facilitating scientific engagement with these stakeholders to develop standardized templates for ethics and regulatory approvals. Through co-developing robust research operating procedures and protocols, these health organizations can substantially streamline the approval process for research initiatives, rendering crucial ethical guidance along the way.

Creating harmonized research frameworks is a strategic move toward presenting a more unified voice in GH, controlling research activities across Africa, and safeguarding against unethical research, particularly in nations with weaker governance and oversight mechanisms. Various stakeholders – like national governments, professional associations, and academic institutions – have made commendable efforts to collaborate on ethics regulatory committees, leading to initiatives like the Pan African Bioethics Initiative, African Bioethics Network, Organisation de Coordination pour la Lutte contre les Endémies en Afrique Centrale, and the African Independent Ethics Committee that was established by the African Social Work Network, among others. However, these efforts often lack the convening authority possessed by the Africa CDC and the AU. This authority is vital for coordinating continent-wide initiatives, fostering intra-African collaboration, responding swiftly to multinational emergencies, mobilizing research resources effectively, and improving both research oversight and regulatory capacity.

## Next steps

**Figure 1.**
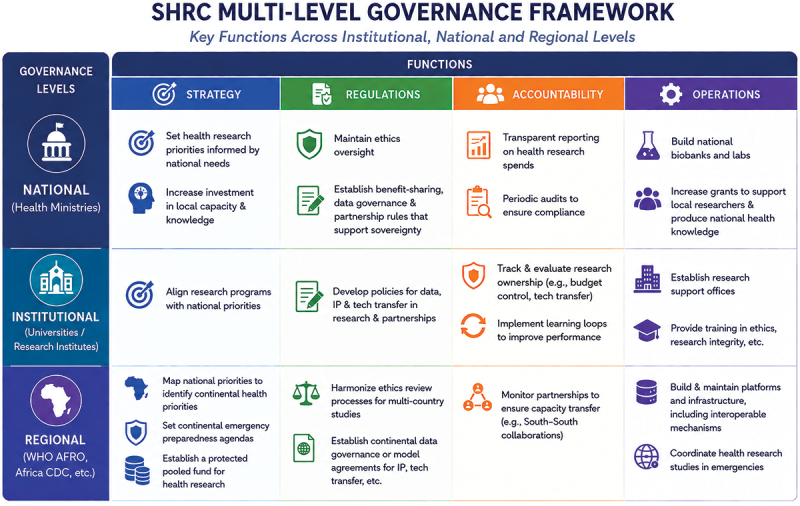
A sovereign, ethical, accountable and collaborative health research ecosystem that delivers impact for Africa.

The SHRC needs to be operationalized through pragmatic governance. For this purpose, a robust multi-level framework – spanning institutional, national, and regional levels – anchored by key functions, particularly strategy, regulations, accountability, and operations, would be required. Figure 1 is a draft scaffold that may be used for further consultations and creating measurable change.

## Data Availability

Data sharing is not applicable to this article as no new data were created or analyzed in this study.
